# Whole-Genome Sequences of Two *Campylobacter coli* Isolates from the Antimicrobial Resistance Monitoring Program in Colombia

**DOI:** 10.1128/genomeA.00131-16

**Published:** 2016-03-17

**Authors:** Johan F. Bernal, Pilar Donado-Godoy, María Fernanda Valencia, Maribel León, Yolanda Gómez, Fernando Rodríguez, Richa Agarwala, David Landsman, Leonardo Mariño-Ramírez

**Affiliations:** a Corporación Colombiana de Investigación Agropecuaria (CORPOICA), Centro de Investigación Tibaítata, Cundinamarca, Colombia; b Instituto Colombiano Agropecuario (ICA), Laboratorio Nacional de Diagnostico Veterinario, Bogotá, Colombia; c National Center for Biotechnology Information, National Library of Medicine, National Institutes of Health, Bethesda, Maryland, USA

## Abstract

*Campylobacter coli*, along with *Campylobacter jejuni*, is a major agent of gastroenteritis and acute enterocolitis in humans. We report the whole-genome sequences of two multidrug-resistance *C. coli* strains, isolated from the Colombian poultry chain. The isolates contain a variety of antimicrobial resistance genes for aminoglycosides, lincosamides, fluoroquinolones, and tetracycline.

## GENOME ANNOUNCEMENT

*Campylobacter* spp. are microaerobic, non-spore-forming, Gram-negative, and oxidase-positive members of the *Campylobacteraceae* family (1). *Campylobacter* spp. are zoonotic pathogens (2) and some of the main bacteria associated with human food-borne illness (3, 4). Campylobacteriosis is frequently associated with the consumption of undercooked poultry meat and the mishandling of the raw poultry products (5, 6). Different *Campylobacter* spp. are recognized as causing human gastroenteritis worldwide (7–9). *Campylobacter coli* is commonly isolated from swine and less so from poultry and humans (7, 9). Although *C. coli* accounts for fewer infections in humans than *Campylobacter jejuni*, its impact is considerable (10), taking into account the increased capability for antimicrobial resistance (11), where multidrug efflux pumps play an important role as mechanisms of antibiotic resistance (3). A small number of studies in Colombia have focused on understanding the epidemiology of *Campylobacter* spp. and their associated antimicrobial resistance. To correct this deficiency, the Colombian Integrated Program for Antimicrobial Resistance Surveillance (COIPARS) (12, 13) has been included in the Colombia-wide *Campylobacter* surveillance program, whose priority is to generate information for different governmental institutions, agricultural enterprises, and food animal production systems. The findings from our studies in Colombia have shown that *C. coli* and *C. jejuni* contamination of raw poultry and poultry meat products present risk factors associated with acute illness in consumers of these products.

Here, we present the whole-genome sequences of two multidrug-resistant *C. coli* strains (M1483 and M1486), isolated from poultry meat collected from two retail stores in Bogotá, Colombia, as part of the COIPARS antimicrobial resistance monitoring program. Genomic DNA was isolated from overnight cultures using the PureLink Genomic DNA minikit (Invitrogen, Grand Island, NY, USA), and DNA libraries were prepared using SureSelect QXT sample preparation kit (Agilent, Santa Clara, CA, USA). The libraries were prepared according to the manufacturer’s instructions and sequenced on an Illumina HiScanSQ instrument with 1 × 151-bp single reads, according to standard Illumina protocols. The *C. coli* M1483 and M1486 genomes were assembled using the reference-guided assembler ARGO, developed at NCBI, and the *de novo* assembler SPAdes (14). The genome sequence of strains M1483 and M1486 consisted of 45 and 55 contigs, yielding total sequences of 1,683,490 bp and 1,780,967 bp, respectively. The overall G+C content of the isolates was determined to be 32%. Sequences were annotated using the NCBI Prokaryotic Genome Automatic Annotation Pipeline (PGAAP) and have been deposited in GenBank. The results of the genome annotation presenting the number of genes, coding sequences, pseudogenes, CRISPR arrays, rRNAs, tRNAs, and noncoding RNAs are summarized in Table 1.

**TABLE 1:** *Campylobacter coli* genome annotation statistics

Strain	NCBI BioSample	No. of genes	No. of CDSs^a^	No. of pseudogenes	No. of CRISPR arrays	No. of rRNAs	No. of tRNAs	No. of ncRNAs^b^	GenBank accession no.
M1483	SAMN04358093	1,782	1,739	55	0	3	37	3	LQXL00000000
M1486	SAMN04358091	1,916	1,873	58	1	3	37	3	LQXK00000000

a CDSs, coding sequences.

b ncRNAs, noncoding RNAs.

A search for resistance-associated genes present in the isolates was performed using ResFinder version 2.1 (15) and enriched using RAST version 2.0 (16), both with default parameters. We found antimicrobial resistance genes for aminoglycosides (Aph 3′-III), lincosamides (*InuC*), fluoroquinolones (*gyrA* and *gyrB*), and tetracyclines (EF-G and TetO). Additionally, we found efflux pump genes (CmeA, CmeB, TolC, MATE, MFS, MacA, MacB, RND, AcrB, and OM) and CmeABCR operon genes, both associated with increased multidrug resistance.

### Nucleotide sequence accession numbers.

This whole-genome shotgun project has been deposited in DDBJ/EMBL/GenBank under the accession numbers listed in Table 1. The versions described in this paper are the second versions.
